# HRG switches TNFR1-mediated cell survival to apoptosis in Hepatocellular Carcinoma

**DOI:** 10.7150/thno.47286

**Published:** 2020-08-20

**Authors:** Xuejing Zou, Dongyan Zhang, Yang Song, Shanshan Liu, Qian Long, Liheng Yao, Wenwen Li, Zhijiao Duan, Dehua Wu, Li Liu

**Affiliations:** 1 Guangdong Provincial Key Laboratory of Viral Hepatitis Research, Hepatology Unit and Department of Infectious Diseases, Nanfang Hospital, Southern Medical University, Guangzhou 510515, China.; 2 State Key Laboratory of Organ Failure Research, Nanfang Hospital, Southern Medical University, Guangzhou 510515, China.; 3 Department of Radiation Oncology, Nanfang Hospital, Southern Medical University, Guangzhou 510515, China.; 4 Department of Medical Quality Management, Nanfang Hospital, Southern Medical University, Guangzhou 510515, China.

**Keywords:** histidine-rich glycoprotein, tumor necrosis factor receptor 1, apoptosis

## Abstract

**Background:** Tumor necrosis factor receptor 1 (TNFR1) signaling plays a pleiotropic role in the development of hepatocellular carcinoma (HCC). The formation of TNFR1-complex I supports cell survival while TNFR1-complex II leads to apoptosis, and the underlying mechanisms of the transformation of these TNFR1 complexes in HCC remain poorly defined.

**Methods:** The interaction protein of TNFR1 was identified by GST pulldown assay, immunoprecipitation and mass spectrometry.* In vitro* and* in vivo* assay were performed to explore the biological features and mechanisms underlying the regulation of TNFR1 signals by histidine-rich glycoprotein (HRG). Data from the public databases and HCC samples were utilized to analyze the expression and clinical relevance of HRG.

**Results:** HRG directly interacted with TNFR1 and stabilized TNFR1 protein by decreasing the Lys(K)-48 ubiquitination mediated-degradation. The formation of TNFR1-complex II was prompted by HRG overexpression via upregulating Lys(K)-63 ubiquitination of TNFR1. Besides, overexpression of HRG suppressed expression of pro-survival genes by impairing the activation of NF-κB signaling in the presence of TNFR1. Moreover, downregulation of HRG was a result of feedback inhibition of NF-κB activation in HCC. In line with the pro-apoptotic switch of TNFR1 signaling after HRG induction, overexpression of HRG inhibited cell proliferation and increased apoptosis in HCC.

**Conclusions:** Our findings illustrate a crucial role for HRG in suppressing HCC via inclining TNFR1 to a pro-apoptotic cellular phenotype. Restoring HRG expression in HCC tissues might be a promising pharmacological approach to blocking tumor progression by shifting cellular fate from cell survival to apoptosis.

## Introduction

TNFR1 is dysregulated in various cancers and is associated with malignant progression. Study showed that TNFR1-mediated signaling cooperates with repressed keratinocyte NF-κB in driving skin cancer development 1. MUC13 interacts with TNFR1 and increases clustering of the TNFR1 signaling complex, thereby amplifying the efficiency of TNF-induced NF-κB activation, which supports colorectal cancer cells to survive under DNA-damaging agents 2. TNFR1 was also noted to be involved in regulating the tumorigenicity of ovarian cancer 3. In liver cancer, TNFR1 is reported to be vital for tumor promotion. TNF-α/ TNFR1 signaling participates in the proliferation of oval cells during the preneoplastic stage of liver carcinogenesis and loss of TNFR1 reduces the incidence of tumor formation 4. TNFR1 signaling contributes to obesity-induced carcinoma promotion as depletion of TNFR1 abolished obesity-enhanced HCC development 5. Based on such tumor-promoting aspects of TNFR1, efforts have been made to target TNF-α or TNFR1 in various malignancies with pharmaceuticals that include TNF-α inhibitors, TNFR1 monoclonal antibodies, and TNFR1-targeting nanomaterials. However, the implements of TNF-α or TNFR1 therapeutic practices are unsatisfactory owing to the dual function and complexity of TNFR1-mediated signaling during cancer development 6-8.

TNFR1 governs either survival or death in cancer cells via formation of different TNFR1-complexes 9,10. Upon TNF-α stimulation, TNFR1 recruits receptor-interacting protein 1 (RIP1) and TNFR1-associated death domain protein (TRADD) to form TNFR1-complex I which increases the expression of a string of pro-survival genes by activating the NF-κB signaling pathway 11,12. Activation of TNFR1-complex I plays a vital role in tumorigenesis as shown in previous studies that investigated the act of TNF-α in the initiation, development, recurrence and therapy resistance of malignancies such as liver cancer, breast cancer, bladder cancer, renal cell carcinoma and pancreatic cancer 13-17. Besides, TNF-α stimulation leads to TNFR1 internalization into the cytoplasm and formation of TNFR1-complex II, also known as death-inducing signaling complex (DISC). Next, TNFR1 recruits fas-associated death domain protein (FADD) and caspase-8 to increase apoptosis, leading to cell death 18. Based on the cell death-promoting effect of TNFR1-complex II, the potency of human recombinant TNF-α as an anti-tumor therapy was tested in numerous clinical trials but declined owing to the unclear role of the TNFR1-mediated signaling pathway in cancer 6. Suppressing NF-κB-dependent living signaling while inducing TNFR1-mediated cell apoptosis is an unresolved conundrum for devising medicines based on TNFR1 modulation.

TNFR1 signaling is regulated by diverse processes that include ubiquitination, glycosylation, endocytosis, lipid raft recruitment, and so on. Among these, ubiquitination is one of the most central post-transcriptional modifications that controls the stability of TNFR1 protein and leads the pro-survival or pro-apoptotic cellular signaling transduction, which determines cancer cell progression 19-22. Given the limited understanding of governing TNFR1-mediated signaling transduction in HCC, we performed this study to explore the regulation of TNFR1 complex I/II and the resulting influence on the cell fate.

Researches demonstrated that histidine-rich glycoprotein (HRG) involves both inflammatory promoting effect in chronic liver disease and tumor suppression during the development of HCC 23-25. HRG was identified as a TNFR1 binding partner in our study. Whether HRG influences the TNFR1 signaling mechanism within cancer cells from a pro-survival to pro-death state has not been explored. A better understanding of the role of HRG in TNFR1-mediated pleiotropic pathways ought to assist in formulating new anticancer therapeutic strategies.

## Materials and Methods

### Patients and tissue samples

HCC samples and matching noncancerous tissues used in our study were collected from HCC patients who underwent hepatectomy between January 2014 and December 2015 at Nanfang Hospital (Guangzhou, China). Patients enrolled did not receive any anti-tumor treatment before surgery. Prior patient consent and approval from the Institute Research Ethics Committee were obtained for the use of the clinical materials for research.

All tissues were stored in a liquid nitrogen container prior to RNA or protein extraction.

### Cell culture

HCC cell lines Huh7 and SMMC-7721 were purchased from the Cell Bank of the Chinese Academy of Sciences (Chinese Academy of Sciences, Shanghai, China). Huh7 and SMMC-7721 cells were incubated in DMEM and RPMI 1640 (Gibco, Grand Island, NY, USA), respectively, with 10% fetal bovine serum (Gibco) at 37 °C in a humidified incubator containing 5% CO_2_. Recombinant human TNF-alpha (PeproTech, Rocky Hill, NJ, USA), FXR agonist GW4064 (Selleck Chemicals, USA), MG132 (Beyotime Biotechnology, China) and cycloheximide (CHX, Sigma-Aldrich, USA) were used to treat cells for experiments.

### Xenograft assays

BALB/c nude mice (4-6 weeks old, Central Laboratory of Animal Science, Southern Medical University, Guangzhou, China) were used for *in vivo* studies. HRG-overexpressed SMMC-7721 cells and the related controls were suspended at a density of 1×10^7^ per 100 μL and inoculated subcutaneously into the flanks of each mouse. After 30 days, the IVIS Lumina II system (Caliper Life Sciences, Hopkinton, MA, USA) was used to measure the fluorescence intensity of tumor before sacrifice. Xenograft tumors were then removed and weighed. Hematoxylin and eosin (H&E), terminal deoxy-nucleotidyl transferase-mediated dUTP nick-end labeling (TUNEL) assay (*In situ* Cell Death Detection Kit, Roche, USA), and immunohistochemical (IHC, Dako, Carpinteria, CA, USA) staining were performed to evaluate the morphology, cell apoptosis, and cell proliferation in all tumor tissues, respectively, according to the manufacturer's guidelines.

### Gene-expression datasets

TCGA cohort: A cohort containing 365 cases of HCC patients with HRG expression data and follow-up information downloaded from The Cancer Genome Atlas Liver Hepatocellular Carcinoma (TCGA-LIHC; https://tcga-data.nci.nih.gov/tcga/) dataset. GSE14520 cohort: A cohort of HCC patients with HRG expression data and follow-up information downloaded from the GEO database (https://www.ncbi.nlm.nih.gov/geo/query/acc.cgi?acc=GSE14520). GSE6764 cohort: 75 samples covering different stages of HCV-induced HCC were analyzed (https://www.ncbi.nlm.nih.gov/geo/query/acc.cgi). Patients were organized according to their HRG expression (from high to low) and then divided into two groups by the median or quartile value. Correlations between HRG expression and overall survival (OS) and progression-free interval (PFI) were analyzed.

### GST pulldown assay

pGEX-6p-1, pGEX-6p-1-GST-TNFR1, and pET-28a-HRG-HIS were transformed into Rosetta (DE3) cells and induced for expression by isopropylthio-β-d-galactoside. The GST pulldown assay was performed according to the manufacturer's instructions (GST pulldown Kit, BersinBio, Guangzhou, China). Briefly, GST-TNFR1 (bait) was purified by using GSH-beads and then incubated with cell lysates containing HRG-His (prey) overnight. The beads were washed and eluted for western blot detection.

### Cell transfection, western blot and immunoprecipitation (IP) analysis, immunofluorescence (IF) assay, dual-luciferase reporter assay, quantitative Real-Time PCR (qRT-PCR), 5-ethynyl-20-deoxyuridine (EdU) assays, flow-cytometry assay and Gene set enrichment analysis (GSEA)

These methods were performed as previously described 26,27 and were detailed in supplementary materials and methods.

### Statistical analysis

All statistical analyses were performed using SPSS 20.0 software (IBM Corp., Armonk, NY, USA). Student's *t*-test or one-way analyses of variance (ANOVA) were used to analyze data of the two groups. Two-way analysis of variance was used for multiple comparisons of different groups. Survival curves and time for OS and PFI were conducted using Kaplan-Meier and log-rank tests. Data were reported as the mean ± SD from three independent experiments unless otherwise noted.* P* values < 0.05 (two-tailed) were considered statistically significant.

## Results

### HRG directly interacts with TNFR1 in HCC cells

**Figure 1:**
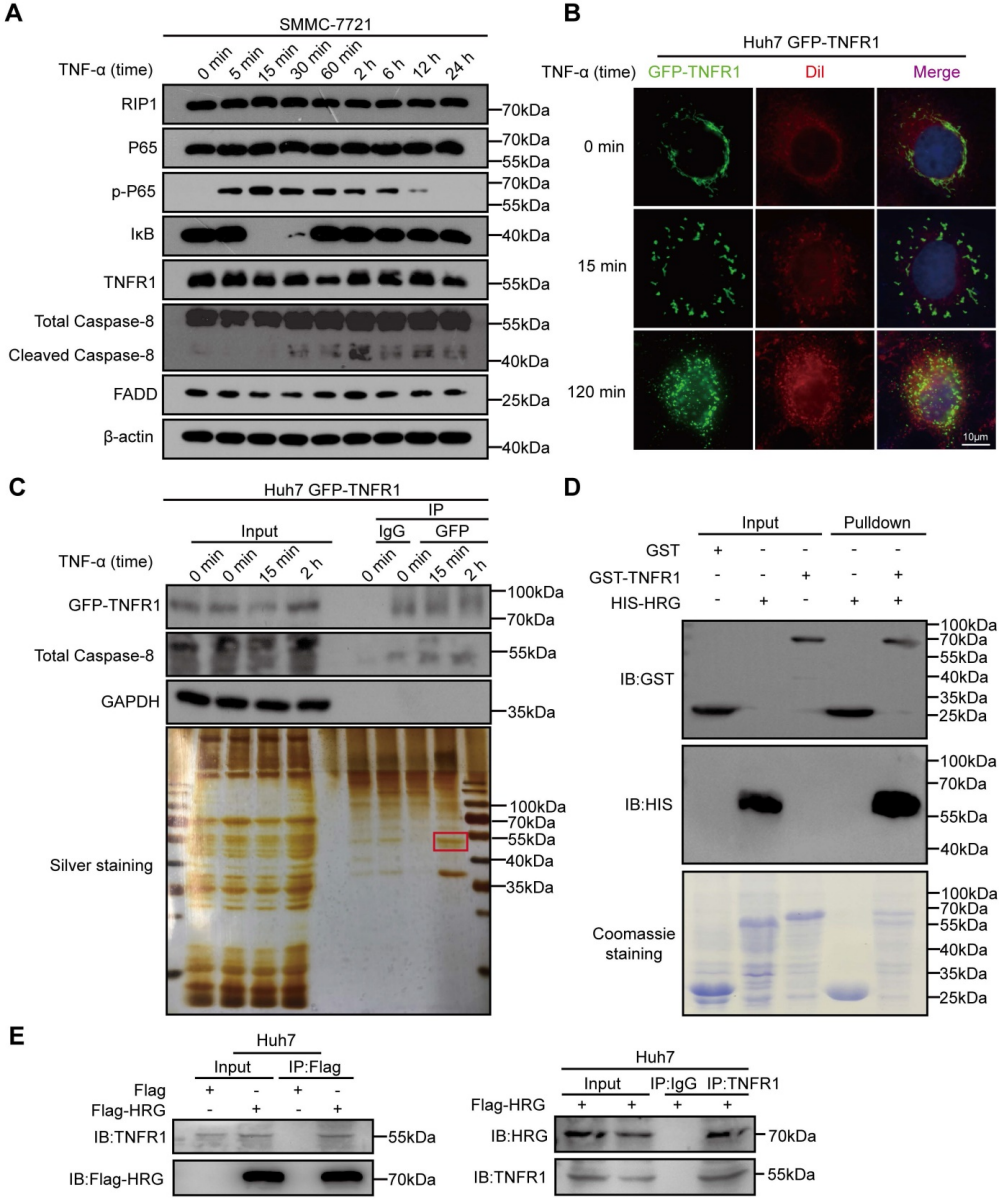
** HRG is a binding partner of TNFR1 in HCC cells. (A)** SMMC-7721 cells were treated with TNF-α for different times, and lysates were then immunoblotted for proteins associated with TNFR1 complexes. **(B)** Green fluorescent protein (GFP)-TNFR1 overexpressing Huh7 cells were seeded and treated with TNF-α (100 ng/ml) for 15 or 120 min, and 0.3 µM Dil was used to stain the cell membrane for 20 min. A fluorescence microscope was used to image the cells (cell membrane, red; GFP, green). **(C)** Top: GFP-TNFR1-overexpressing Huh7 cell lysates were precipitated with magnetic beads conjugated with GFP and immunoblotted for GFP-TNFR1, Caspase-8, and GAPDH. Bottom: silver staining. **(D)** A direct interaction between GST-TNFR1 and HIS-HRG was detected in GST pulldown experiments. Western blot analysis of GST or HIS-tagged proteins of GST-input and pulldown samples. **(E)** Western blot analysis of whole cell lysate and co-immunoprecipitated samples of IgG, anti-Flag, or anti-TNFR1 were obtained from Huh7 cells 48 h after transfected with the indicated plasmids.

In order to investigate the signals representing different TNFR1-complexes under TNF-α stimulation in HCC cells, we detected effectors of TNFR1-complex I (phopsho-P65/P65 and IκB) and TNFR1-complex II (cleaved Caspase-8/Caspase-8 and FADD) in SMMC-7721 cells exposed to TNF-α via western blotting. We found that peak expression of phospho-P65 that indicated the formation of TNFR1-complex I and activation of the NF-κB signaling pathway occurred 15 min after TNF-α stimulation. Apoptosis inducers cleaved Caspase-8 peaked 2 h after TNF-α treatment, suggesting that TNFR1 transformed from complex I to complex II (Figure 1A and Figure S1A). To further trace the transformation of the TNFR1 complex from 15 min to 2 h after TNF-α treatment, we applied IF assay and detected the cellular localization of GFP-TNFR1. IF assay images of Huh7 cells showed that TNFR1 was bound to the cytomembrane before TNF-α exposure and translocated to the cytoplasm 2 h later (Figure 1B). We next investigated the mechanism that favored the conversion of TNFR1-complex II. A co-IP assay followed by mass spectrometry was performed to identify TNFR1 interactors within 2 h of TNF-α stimulation of Huh7 cells (Figure 1C and Table S1). Nine proteins including HRG were found in the common between the mass spectrometry data and interactors of TNFR1 predicted from the Biogrid database (Figure S1B, C). Furthermore, analysis of TCGA showed that HRG mRNA expression was the most favorable prognostic marker in liver cancer and positively correlated with TNFR1 mRNA levels (Figure S1D-E). To confirm their association, we performed GST pulldown assay and co-IP experiments, and verified that TNFR1 directly bound to HRG (Figure 1D, E). Collectively, these data indicate that HRG is physically associated with TNFR1 and may be a regulator of TNFR1-mediated downstream signaling.

### HRG promotes the formation of TNFR1-complex II

**Figure 2:**
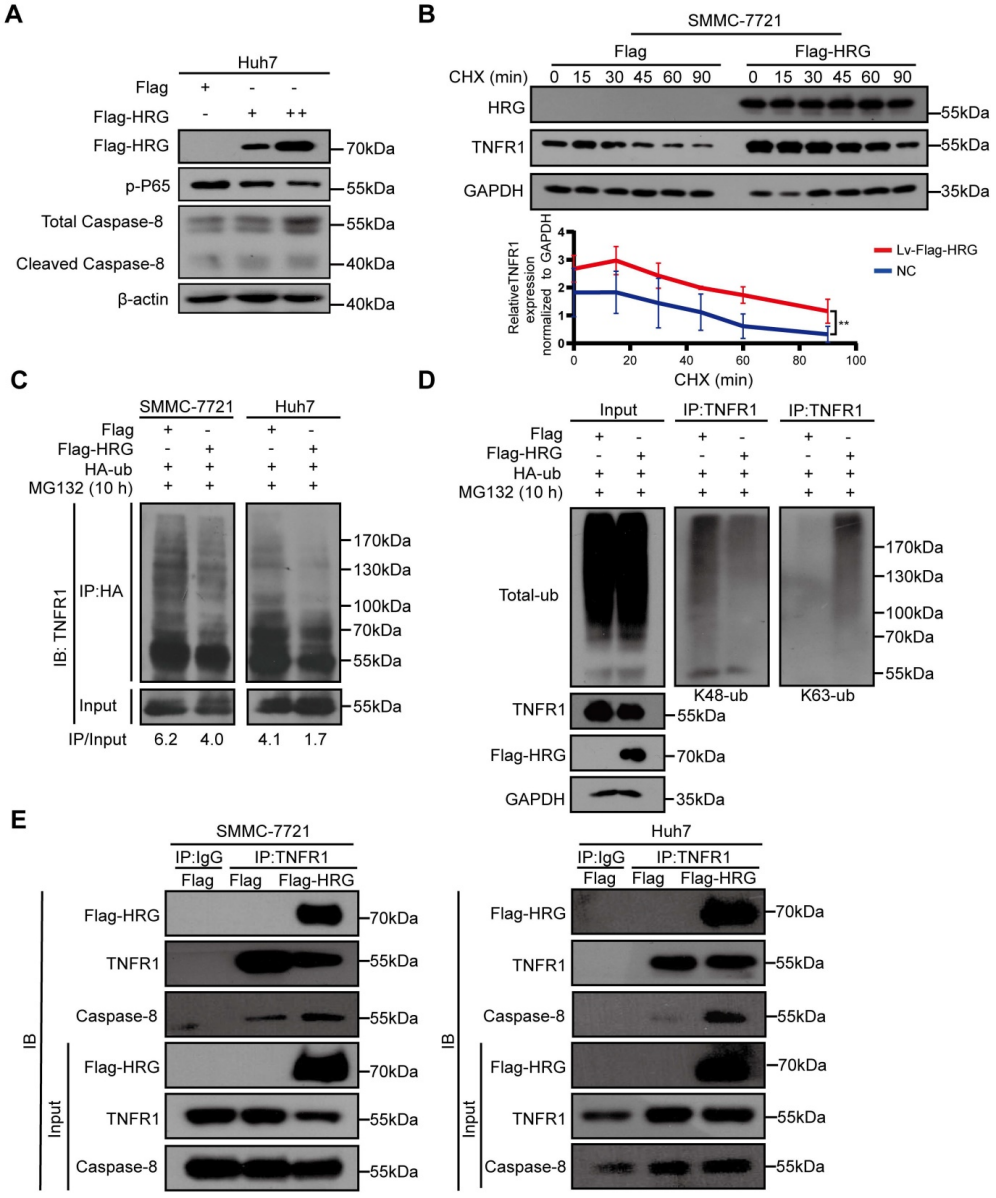
** HRG increases the stability of the TNFR1 protein and promotes the formation of TNFR1-complex II in HCC cells. (A)** Huh7 cells transfected with concentration-dependent Flag-HRG plasmid and immunoblotted for proteins associated with TNFR1 complexes. **(B)** Protein stability of TNFR1 was determined by collecting the protein samples at 0, 15, 30, 45, 60, and 90 min in the presence of cycloheximide (100 µg/mL) in SMMC-7721 HRG-overexpressing cells and controls. **(C)** HRG-overexpressing SMMC-7721 and Huh7 cells and their controls were transfected with hemagglutinin (HA)-tagged ubiquitin (Ub) expression vector for 48 h. The HA immunoprecipitation products or whole cell lysates were analyzed by western blotting. **(D)** Total and K48/K63-specific ubiquitination were detected in SMMC-7721 HRG-overexpressing cells and controls after HA-Ub transfection (48 h) and MG132 treatment (10 µM). **(E)** HRG-overexpressing SMMC-7721 and Huh7 cells and their controls were precipitated with magnetic beads conjugated to TNFR1, and blotted for Flag-HRG and Caspase-8.

Based on the above findings, we hypothesized that HRG induced TNFR1 to form TNFR1-complex II in HCC cells. We therefore transfected increasing doses of Flag-HRG plasmid into Huh7 cells and measured the signals of TNFR1 complex I/II using western blotting. We found that Caspase-8, and cleaved Caspase-8 were upregulated while phospho-P65 was gradually reduced as HRG levels increased (Figure 2A). Also, we found that TNFR1 showed a longer half-life in cells stably overexpressing HRG than in control cells after CHX treatment (Figure 2B). The mRNA levels of TNFR1 in HRG stable overexpressing SMMC-7721 and Huh7 cells remained unchanged when compared to control cells as measured by qRT-PCR, suggesting that HRG regulates TNFR1 in a post-transcriptional manner (Figure S2A-C).

As protein stability is mainly controlled by ubiquitination 28,29, we hypothesized that HRG maintained TNFR1 expression by reducing the ubiquitination-mediated degradation of TNFR1. We found that TNFR1 ubiquitination levels in HCC cells stably overexpressing HRG were lower than those in control cells, suggesting that overexpression of HRG inhibits the total ubiquitination level of TNFR1 (Figure 2C). Studies have shown that Lys(K)-48 ubiquitination mediates protein degradation, and that Lys(K)-63 ubiquitination of TNFR1 promotes its internalization and complex II formation 30. Further exploration into the K48 and K63 ubiquitination levels of TNFR1 found that overexpression of HRG reduced the level of K48 ubiquitination which influenced protein degradation, and increased the level of K63 ubiquitination of TNFR1 (Figure 2D and Figure S2D). To identify the pivotal E3 ubiquitin ligase that triggered these progress, we first compared the Lys(K)-63 ubiquitination level of TNFR1 between HRG overexpression and control samples in the absence of previously reported E3 ubiquitin ligases (RNF8 and TRAF6) of TNFR1 30,31. We found that upregulation of Lys (K)-63 ubiquitination of TNFR1 by HRG was RNF8- and TRAF6-dependent (Figure S2E). Furthermore, LC-MS analysis of TNFR1 co-IP lysates of different groups (control, TNF-α 15 min stimulation, TNF-α 2 h stimulation, and HRG overexpression) showed that UHC-L1, a C-terminal ubiquitin hydrolase, decreases the Lys (K)-63 ubiquitination of TNFR1 and disassociated from TNFR1 in the TNF-α 2 h stimulation and HRG overexpression groups compared to that in the control and TNF-α 15 min stimulation groups (Figure S2F and Table S2). Overall, HRG may disrupt the interaction between TNFR1 and UHC-L1, thereby increasing RNF8- or TRAF6-mediated Lys (K)-63 ubiquitination of TNFR1.

In line with upregulation of Lys (K)-63 ubiquitination, HRG increased the binding of TNFR1 to Caspase-8, indicating that HRG promoted the formation of TNFR1-complex II (Figure 2E). Taken together, these findings showed that the overexpression of HRG increases the stability of TNFR1 protein and promotes the formation of TNFR1-complex II.

### HRG inhibits NF-κB signaling pathway activation

**Figure 3:**
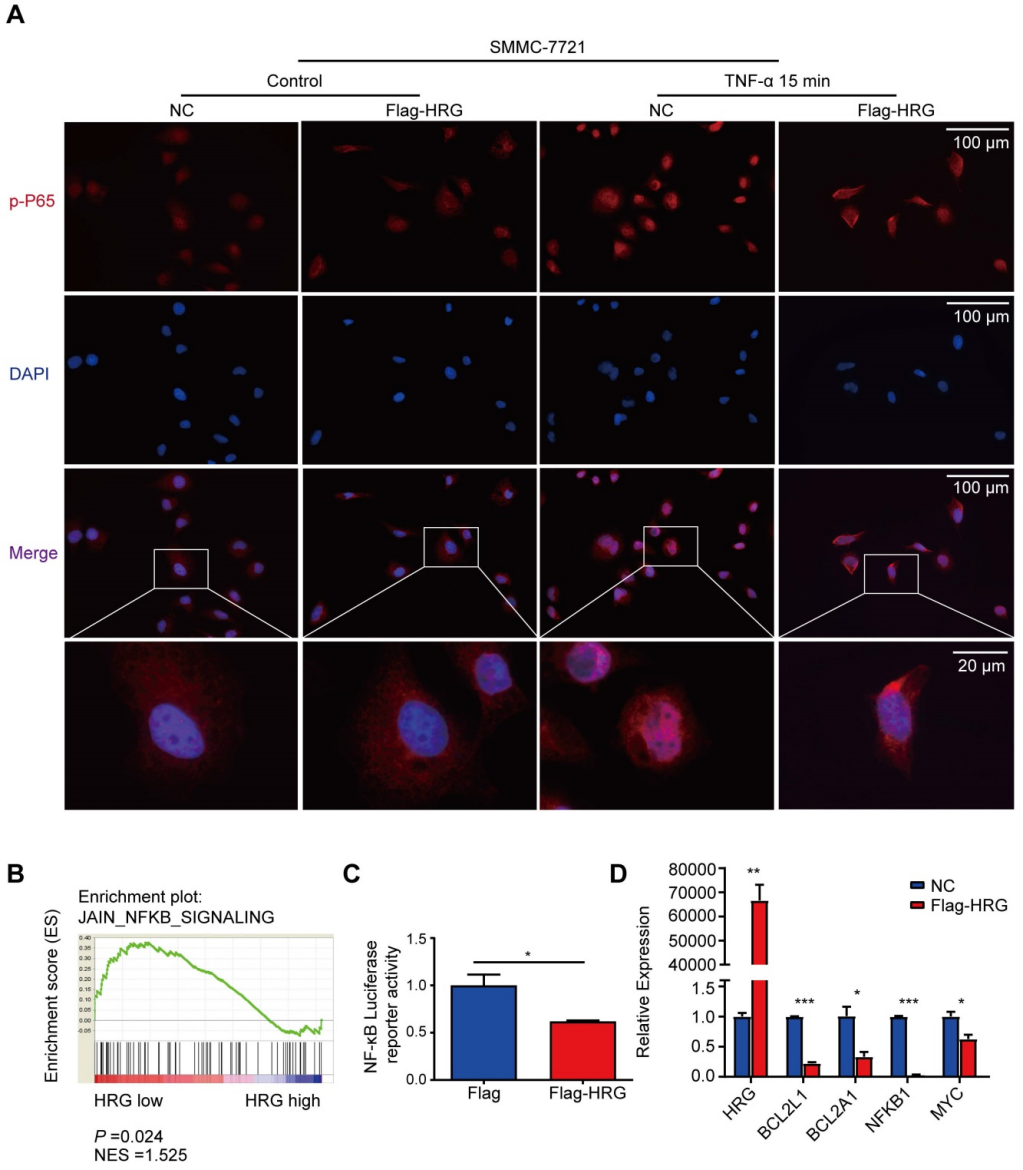
** HRG inhibits the NF-κB signaling pathway in HCC cells. (A)** SMMC-7721 cells were stably transfected with Flag-HRG lentivirus and control vector and treated with TNF-α for 15 min, and were then analyzed by immunofluorescence for phospho-P65 (red) and DAPI (blue). **(B)** Results of gene set enrichment analysis (GSEA) were plotted to visualize the correlation between the expression of HRG and gene signatures of NF-κB signaling in TCGA cohort. **(C)** The activity of NF-κB was determined using a luciferase assay in Flag-HRG overexpressing and control SMMC-7721 cells. **(D)** mRNA expression of *BCL2L1*, *BCL2A1*, *NFKB1*,* MYC*, and *RELA* were detected by quantitative reverse transcription PCR in Flag-HRG overexpressing and control SMMC-7721 cells. (*, *P* < 0.05; **, *P* < 0.01; ***, *P* < 0.001).

Since our data suggest that HRG promotes TNFR1-complex II formation, we next ask if HRG regulates the NF-κB signaling pathway, which may be activated to transcript its anti-apoptotic target genes to suppress TNFR1-complex II induced apoptosis. Immunofluorescence experiments showed that P65 was phosphorylated and underwent nuclear translocation within 15 min of TNF-α stimulation in SMMC-7721 control cells, whereas HRG overexpression inhibited this process (Figure 3A). Meanwhile, HRG knockdown promoted the nuclear translocation of phospho-P65 (Figure S3A). Furthermore, GSEA performed on liver cancer samples in the TCGA database implied that low expression of HRG was positively associated with NF-κB signaling (Figure 3B). We therefore utilized an NF-κB luciferase reporter system and found that HRG suppressed NF-κB luciferase activity (Figure 3C). Next, the expression of *BCL2L1*, *BCL2A1*, *NFKB1* and *MYC* mRNA were evaluated in HRG-overexpressed SMMC-7721 cells and its controls to determine whether HRG regulates NF-κB-modulated pro-survival genes. As expected, upregulation of HRG significantly decreased the expression of these genes (Figure 3D). Furthermore, co-expression analyses using ChIPBase v2.0 showed that HRG was negatively correlated with *RELA*, *BCL2L1*,* CFLAR*, and* NFKB1* expression (Figure S3B) 32, further suggesting that HRG is a negative regulator of the NF-κB signaling pathway.

### HRG inhibits HCC cell proliferation and promotes cell apoptosis

**Figure 4:**
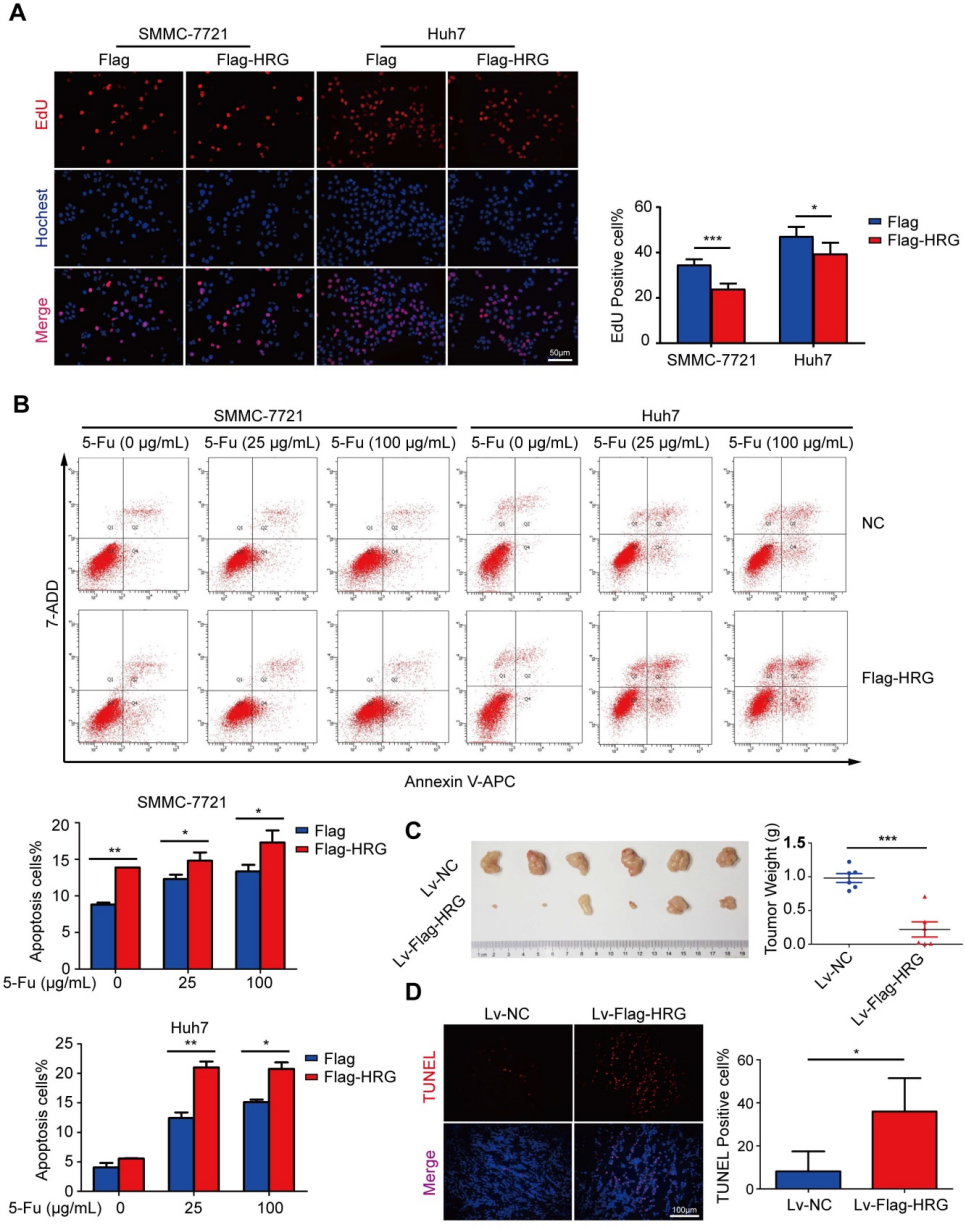
** HRG overexpression suppresses proliferation and promotes apoptosis *in vitro* and *in vivo*. (A)** The effect of HRG overexpression on SMMC-7721 and Huh7 cells was determined using EdU assays. **(B)** Apoptosis among HRG-overexpressing SMMC-7721 and Huh7 cells and their controls as induced by 25 or 100 µg/mL of 5-fluorouracil was detected using flow cytometry. **(C)** Left: HRG-overexpressing and control SMMC-7721 cells derived from subcutaneous neoplasms are shown (n=6); Right: statistical plot of the average tumor weights from the subcutaneous xenograft tumor model. **(D)** TUNEL staining (red) was used to detect apoptotic cells in HRG-overexpressing and control SMMC-7721 cells derived from subcutaneous tumor tissues. (*,* P* < 0.05; ***, *P* < 0.001).

The above results indicated that HRG promoted the formation of TNFR1-complex II and inhibited the activation of the NF-κB signaling pathway, which strongly suggests that HRG plays a role as a tumor suppressor in HCC. To evaluate the biological function of HRG in HCC, Flag-HRG was stably overexpressed in HCC cells via lentiviral transduction (Figure S2C). EdU assays indicated that the proliferation of HRG-overexpressing HCC cells was slower than that of controls (Figure 4A), whereas HRG knockdown enhanced the proliferation of HCC cells (Figure S4A). Recent investigations have noted the post-translational modification of the TNFR1 as a potential anticancer therapeutic strategy 33. Our work identified that HRG regulated the TNFR1 in a post-translational manner. Therefore, we sought to determine the effect of HRG on 5-fluorouracil-induced apoptosis in HCC cells. FACS analysis displayed a significantly higher percentage of annexin V-positive cells in the HRG-overexpressing group than in controls (Figure 4B). Consistent with our *in vitro* data, xenograft tumors derived from cells overexpressing Flag-HRG presented lower weights and *in vivo* luciferase activity than vector-transduced controls (Figure 4C and Figure S4B). TUNEL assays showed a significantly higher rate of apoptosis in Flag-HRG-overexpressing xenograft tumors than in controls (Figure 4D). Additionally, levels of the Ki-67 proliferation marker were markedly reduced in xenograft tumors developed from Flag-HRG-overexpressing cells compared with the controls (Figure S4C, D). Collectively, these results demonstrated that HRG inhibits HCC cell proliferation and promotes apoptosis both *in vitro* and *in vivo*.

### HRG exerts its biological function via TNFR1

**Figure 5:**
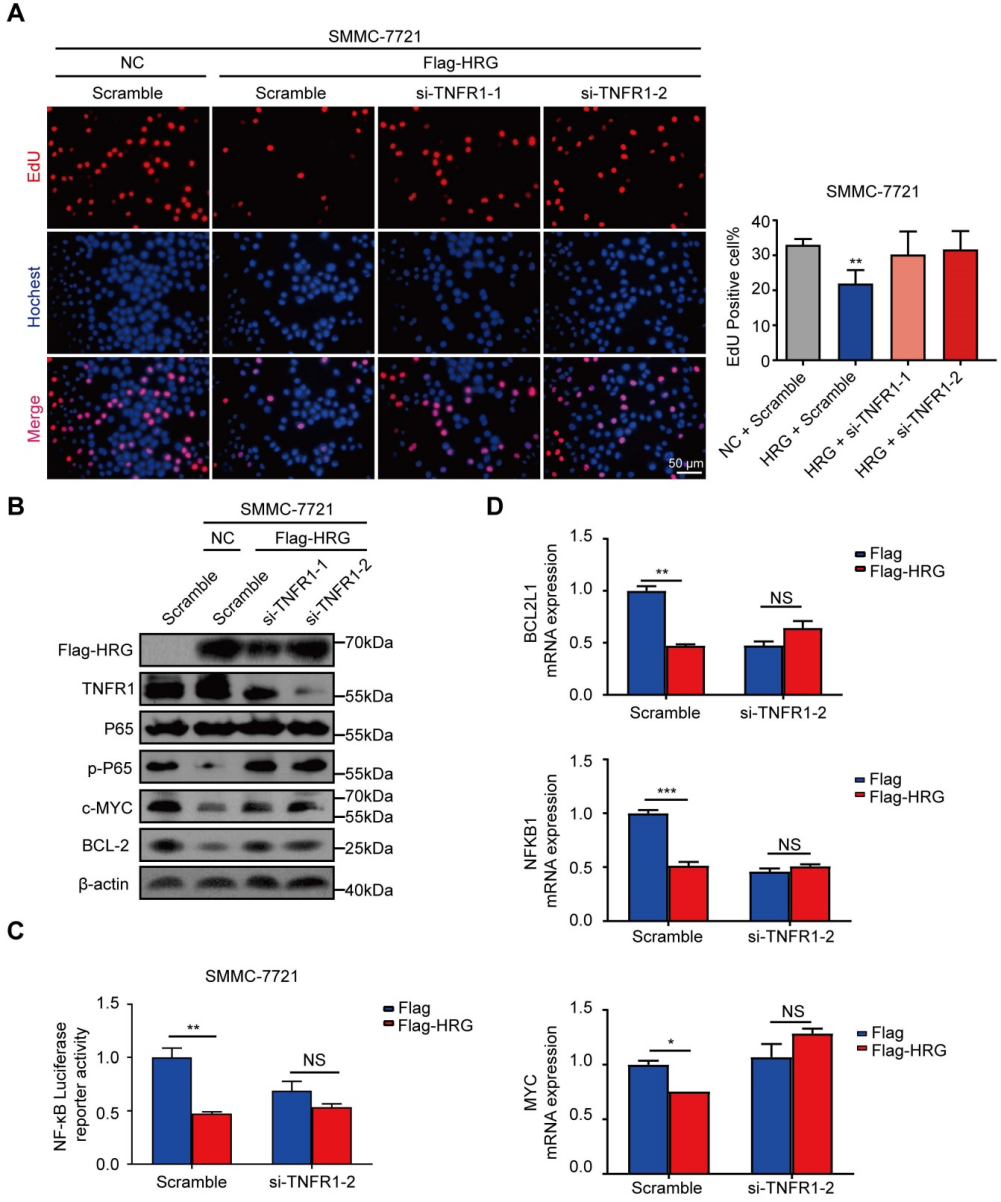
** TNFR1 mediates the biological functions of HRG in HCC cells. (A)** SMMC-7721 cells were co-transfected with Flag-HRG and TNFR1 siRNA, following which cell proliferation was detected via EdU assays. **(B)** The expression of phospho-P65, c-MYC, and BCL-2 was detected by western blotting after TNFR1 was silenced and HRG was overexpressed in SMMC-7721 cells. **(C)** The activity of NF-κB was determined using a luciferase assay in SMMC-7721 cells after transfection with Flag-HRG plasmids and TNFR1 siRNA. **(D)** The mRNA expression levels of *BCL2L1*, *NFΚB1*, and *MYC* in SMMC-7721 were detected using qRT-PCR after the cells were transfected with Flag-HRG plasmids and TNFR1 siRNA. (*, *P* < 0.05; **, *P* < 0 0.01; ***,* P* < 0.001; NS = not significant).

Given that HRG promoted the formation of TNFR1-complex II and inhibited NF-κB signaling activation, we next pursued to verify whether HRG functions through regulating TNFR1. The suppression of proliferation by HRG was reversed when TNFR1 was silenced using siRNA (Figure 5A). The suppressive effect of HRG overexpression on NF-κB signaling was also reversed when combined with TNFR1 knockdown in SMMC-7721 cells (Figure 5B). Consistent with these findings, the inhibitory effect of HRG on NF-κB-luciferase reporter activity was TNFR1-dependent (Figure 5C). The expression levels of NF-κB target pro-survival genes, which were decreased in the HRG-overexpressing group, were restored with the simultaneous knockdown of TNFR1 (Figure 5D and Figure S5A), indicating that the inhibitory effect of HRG on NF-κB signaling is TNFR1 dependent. Moreover, cellular apoptosis induced by HRG overexpression was also dependent on the presence of TNFR1 (Figure S5B). These data suggest that the mediating role of HRG on cell proliferation and apoptosis in HCC cells is executed, at least in part, by TNFR1.

### HRG is downregulated in HCC tissues and inhibited by NF-κB

**Figure 6:**
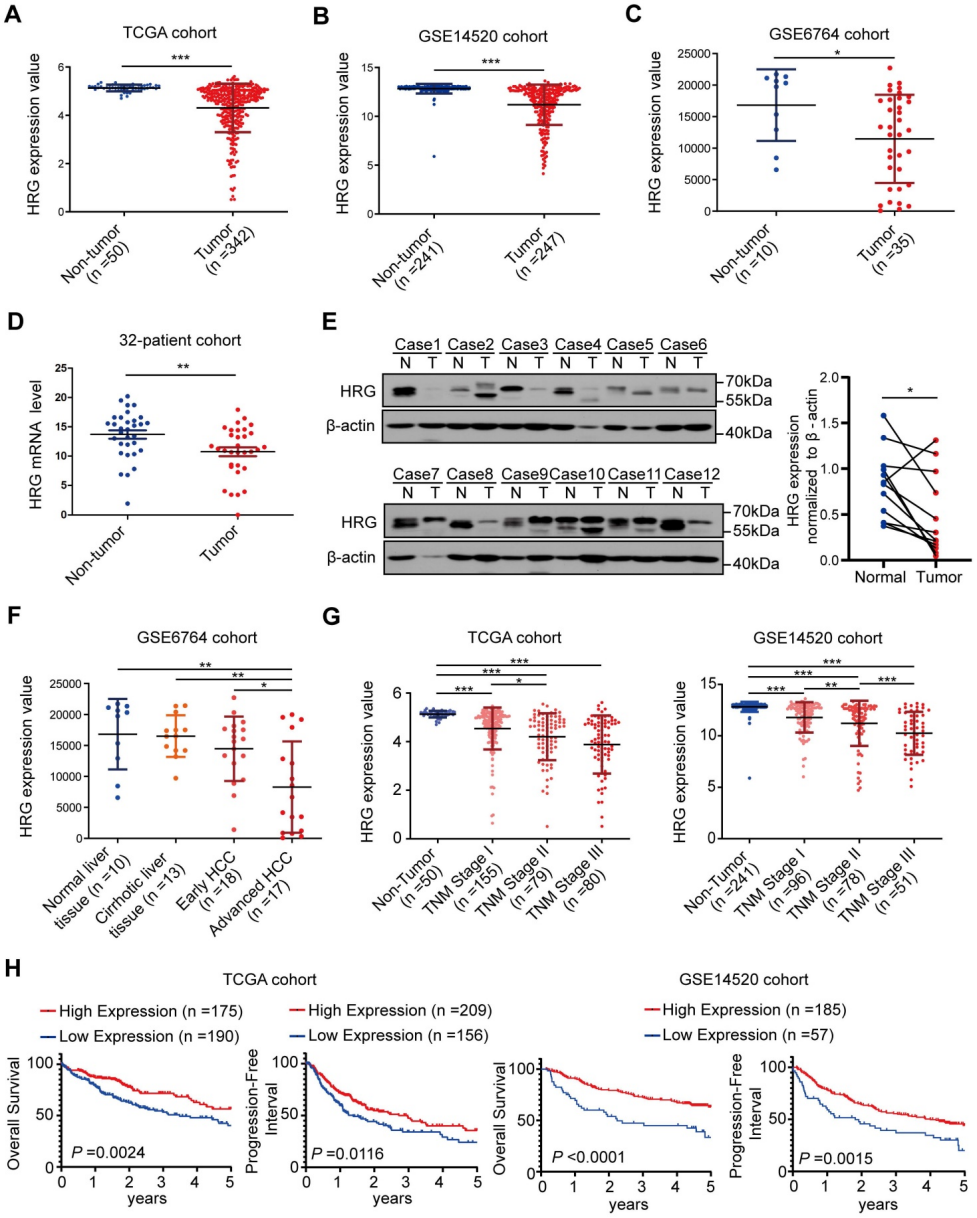
** HRG expression is down-regulated in HCC and is correlated with prognosis. (A)** Expression of HRG in TCGA cohort (including 342 and 50 tumor and non-tumor samples, respectively). **(B)** Expression of HRG in the GSE14520 cohort (247 and 241 tumor and non-tumor samples, respectively).** (C)** Expression of HRG in the GSE6764 cohort (35 and 10 tumor and non-tumor samples, respectively). **(D)** qRT-PCR detection of HRG expression in tumor tissues and adjacent non-tumor tissues in a 32-patient cohort. **(E)** Western blot analysis was used to detect the expression of HRG protein in 12 pairs of hepatocellular carcinoma (HCC) tissues (T) and their matched normal tissues (N). **(F)** HRG expression in various patients clinically diagnosed with HCC were analyzed from the GSE6764 cohort (normal liver, n=10; cirrhotic liver tissue, n=13; early HCC, n=18; and advanced HCC, n=17).** (G)** HRG expression from different patients with HCC were analyzed from TCGA cohort (non-tumor, n=50; stage I, n=155; stage II, n=79; and stage III, n=80) and the GSE14520 cohort (non-tumor, n=241, stage I, n=96, stage II, n=78, and stage III, n=51).** (H)** Kaplan-Meier analysis of overall survival and progression-free interval in TCGA and GSE14520 cohorts based on HRG expression status. X-tile software was used to generate optimal cut-off values and separate patients into high and low HRG expression groups. (*, *P* < 0.05; **, *P* < 0 0.01; ***, P < 0.001).

Our previous microarray analysis revealed that expression of HRG was downregulated in HCC tissues when compared with paired non-tumorous samples (n=7, *P*=0.006; fold change = 29.56) 27. Furthermore, expression data from the TCGA, GSE14520, and GSE6764 datasets showed that HRG was dramatically lower in tumor tissues than in non-tumorous counterparts (Figure 6A-C). Downregulation of HRG in HCC tumor samples was confirmed by qRT-PCR (32 paired HCC samples; Figure 6D) and western blotting (12 matched HCC tissues; Figure 6E).

We then explored the mechanism of HRG downregulation in HCC tumor samples, where pan-cancer alteration frequency analysis revealed no homozygous HRG deletions (Figure S6A). Reduced gene expression could also result from promoter methylation or inhibitory transcription factor interference. Indeed, we observed a strong correlation between DNA methylation and HRG mRNA expression using the cBio cancer genomics portal (Figure S6B) 34. Previous studies had found that HRG was a transcriptional target gene of nuclear bile acid receptor Farnesoid X receptor (FXR) in liver cells 35. NF-κB binds to FXR and subsequently suppresses its transcriptional activity, leading to the downregulation of FXR downstream genes 36,37. According to previous studies, HRG mRNA expression might be inhibited by NF-κB activation via the suppression of FXR. To investigate this, we measured HRG mRNA expression in P65-overexpressing SMMC-7721 cells and found it to be downregulated (Figure S6C-D). Moreover, we found that HRG expression was suppressed when Huh7 cells were treated with TNF-α alone, suggesting that NF-κB activation inhibited HRG transcription. FXR agonist increased HRG mRNA expression in Huh7 cell, which was reversed by adding TNF-α; this supported our hypothesis that HRG was reciprocally inhibited by NF-κB via FXR (Figure S6E). Taken together, our results confirmed that HRG was downregulated in HCC tissues and underwent both DNA methylation and negative feedback inhibition following NF-κB activation.

### HRG expression diminishes with cancer progression, and low expression of HRG correlates with poor prognosis in patients with HCC

Information from GSE6764 datasets showed that expression of HRG decreased progressively from normal liver tissues to advanced HCC (Figure 6F). Moreover, HRG expression in samples that were clinically diagnosed as non-tumorous as well as those representing stages I, II, and III HCC were extracted from TCGA-LIHC and GSE14520 datasets and analyzed. HRG levels decreased as HCC developed and progressed (Figure 6G). GSEA analysis of the TCGA cohort showed that low expression of HRG was positively associated with the gene signatures of poor prognosis (Figure S7A-B). Additionally, survival analysis of TCGA and GSE14520 cohorts showed that patients with high HRG expression exhibited improved overall survival and progression- free interval (Figure 6H and Table S3). These data signified that low HRG expression correlates with worse HCC prognosis. In all, our study revealed that HRG is a regulator of TNFR1 that promotes the formation of TNFR1 complex II, which shifts cancer cells from pro-survival to pro-death states. HRG expression in HCC is suppressed by DNA methylation or via feedback inhibition by NF-κB, thereby supporting cancer cells to survive and thrive (Graphical abstract).

## Discussion

We demonstrated for the first time that HRG interacts with TNFR1, thus promoting the formation of TNFR1-complex Ⅱ and restraining the activation of NF-κB signaling to suppress HCC. Nonetheless, HRG expression is limited in HCC by negative feedback loop to prevent TNFR1- dependent apoptosis. Accordingly, our investigation revealed that restoring HRG expression in HCC cells might be a new approach to inhibiting tumor progression.

The behaviors of TNFR1 in the advancement of HCC are paradoxical. In particular, it has been demonstrated that tumorigenesis was reduced in TNFR1-knockout mice 4, suggesting that TNFR1 functions as a tumor promotor in liver cancer. In contrast, TNFR1 promoter -329G/T polymorphism resulting in allele-specific repression of TNFR1 expression is reported to be associated with HCC 38, indicating that TNFR1 acts as a tumor suppressor. Besides, study has shown that TNFR1 had no significant effect on DEN induced hepatocarcinogenesis model 39. Additionally, patients with high TNFR1 mRNA expression levels showed worse overall survival in TCGA cohort, but no significant difference in GSE14520 cohort (Figure S7C-D). These conflicts may be due to the pleiotropism of TNFR1-mediated pathways. Our study found that HRG promoted TNFR1-complex Ⅱ formation while downregulating the NF-κB activity. These data provide evidence for the dual effects of TNFR1-mediated pathways are controlled by HRG. Therefore, the present work may explain the cancer-promoting effect of TNF-α/TNFR1 in that 9,40, upon TNF-α stimulation, cancer cell signaling pathways shift towards TNF-α/TNFR1-mediated anti-apoptotic signals owing to low HRG expression, and thus to pro-tumor progression and drug resistant states.

According to *in vitro* and* in vivo* experiments, HRG is critical for TNFR1-induced cellular apoptosis in HCC cells. On the one hand, HRG restricted nuclear translocation of NF-κB upon TNF-α treatment, inhibited downstream anti-apoptotic genes, and reduced TNFR1-mediated cell proliferation. The NF-κB signaling repressing effect of HRG is supported by a recent study that HRG inhibits the activation of pro- inflammatory signaling such as NF-κB 41. On the other hand, HRG increases interaction between TNFR1 and Caspase8, which forms TNFR1-complex Ⅱ and directly leads cell apoptosis. Similarly, cellular apoptosis induced by HRG has been documented in fibrosarcoma 42. Thus, we propose a pivotal role for HRG as a molecular switch inclining TNFR1 mediated-survival to apoptosis.

Our study found that TNFR1 expression is upregulated by HRG overexpression. Cellular TNFR1 level is reported to be correlated with sensitivity to MLN4924/TNF-induced cell death in multiple myeloma 43. Increased sensitivity of HCC cells to 5-fluorouracil-induced apoptosis was found with HRG overexpression which enhanced TNFR1 protein stability in the present study, pointing out a possibility that ways to upregulate HRG expression may become an adjuvant approach for cancer treatment. It occurs to our mind that way to recover HRG expression in HCC cells would suppress HCC progression, which was confirmed by experiments performed in HCC cells and nude mice model. Moreover, it is possible that restoring HRG levels in non-immunodeficient mice or patience may exert a better anti-tumor effect owing to its ability to “reeducate” the tumor microenvironment. It is well known that HRG promotes the transformation of M2 tumor-associated macrophages to the M1 phenotype, which inhibits tumor angiogenesis and cell proliferation 44,45. Additionally, it was recently discovered that HRG increased natural killer (NK) cell cytotoxicity against K562 cells by modulating programmed cell death 1 (PD-1) expression 46. Plus, TNFR1 is widely distributed on the surfaces of various immune system-related cell types such as macrophages and regulatory T cells 47,48. Further investigation regarding whether HRG influences immune or other cells expressing TNFR1 may lead to revealing novel mechanisms in cancer development for which new therapeutic approaches can be devised.

The idea of how to increase HRG level in HCC prompted us to investigate the nature of HRG downregulation in tumor samples and methods to restore its expression. Our study showed that the expression of HRG in HCC samples is extremely low and decreased HRG expression was associated with worse HCC prognosis. Moreover, we found that downregulation of HRG was a result of DNA methylation and feedback inhibition of NF-κB activation. Previous studies have shown that NF-κB interacts with FXR and inhibits its transcriptional activity 49,50. HRG is reported to be a direct transcriptional target of FXR in hepatocytes, and FXR agonists can effectively upregulate serum HRG levels in mice and healthy human volunteers 35. Enlightened by these studies, we treated HCC cells with FXR agonists and found that it upregulated HRG mRNA and protein expression in cells and increased their sensitivity to apoptosis induced agent (Figure S6E-F). Herein, we proposed that FXR agonists may contribute to switching from TNFR1-mediated cancer cell survival signaling to pro-apoptotic states, and combined treatments with FXR agonists and pro-apoptotic drugs may produce more potent anti-tumor effects in HCC.

## Conclusions

Collectively, our data elucidated a crucial role for HRG as a molecular switch for directing TNFR1-complex I versus II, thereby promoting pro-apoptotic pathways and suppressing HCC progression. As tumors progress, HRG expression is suppressed by NF-κB activation and continuous TNF-α that is produced in an autocrine fashion or introduced via exogenous stimulation. In this situation, TNFR1-complex I forms and promotes NF-κB pro-survival signaling given the lack of inhibition by HRG, thereby maintaining HCC malignancy. In contrast, inducing HRG breaks the vicious cycle of tumor progression by enhancing TNFR1-complex II formation. Impairment of NF-κB signaling reverses HRG suppression, which further increases TNFR1-induced cell apoptosis. Therefore, upregulating the HRG-TNFR1-pro-apoptosis pathway may be a promising therapeutic approach for patients with HCC.

## Supplementary Material

Supplementary figures.

Supplementary table S1.

Supplementary table S2.

Supplementary table S3.

Supplementary table S4.

## Abbreviations

HRG: Histidine-rich glycoprotein
TNFR1: Tumor necrosis factor receptor 1
HCC: Hepatocellular carcinoma
RIP1: Receptor-interacting protein 1
TRADD: TNFR1-associated death domain protein
DISC: Death-inducing signaling complex
FADD: Fas-associated death domain protein
FXR: Farnesoid X receptor
CHX: Cycloheximide
H&E: Hematoxylin and eosin
TUNEL: Terminal deoxy-nucleotidyl transferase-mediated dUTP nick-end labeling
IHC: Immunohistochemical
TCGA: The Cancer Genome Atlas
GEO: Gene Expression Omnibus
OS: Overall survival
PFI: Progression-free interval
GSEA: Gene set enrichment analysis
IP: Immunoprecipitation
IF: Immunofluorescence
qRT-PCR: Quantitative Real-Time PCR
EdU: 5-ethynyl-20-deoxyuridine
ANOVA: One-way analysis of variance
BCL2L1: Bcl-2-like protein 1
BCL2A1: BCL2 Related Protein A1
NFKB1: Nuclear Factor Kappa B Subunit 1
MYC: MYC Proto-Oncogene
RELA: RELA Proto-Oncogene
CFLAR: CASP8 And FADD Like Apoptosis Regulator
