# Supplementary material for: HRG switches TNFR1-mediated cell survival to apoptosis in Hepatocellular Carcinoma
Source: Theranostics. 2020 Aug 20;10(23):10434–47. doi: 10.7150/thno.47286 (PMC7482824; doi:10.7150/thno.47286)
Supplement: Supplementary file 1 — Supplementary figures. [file thnov10p10434s1.pdf]

HRG switches TNFR1-mediated cell survival to apoptosis in hepatocellular carcinoma

Supplementary

Materials and methods

Cell transfection

The lentiviral vector containing human HRG (NM_000412) was purchased from Genechem Company Ltd (Shanghai, China). Lentiviral vectors containing the HRG gene sequence or the empty vector were introduced into Huh7 and SMMC-7721 cells according to the manufacturer's instruction. The cells were treated with puromycin (1 μg/mL, GeneChem, Shanghai, China) for 2 weeks for stable selection. Small interfering RNAs (siRNAs) were used to silence human HRG, TNFRSF1A, RNF8 and TRAF6 genes expression using Lipofectamine RNAiMAX Transfection Reagent (Invitrogen, Carlsbad, CA, USA) according to the manufacturer's instruction. The siRNAs were synthesized by Genepharma Company (Suzhou, China) and the sequence details were listed in Table S4.

Western blotting and immunoprecipitation analysis

Total protein was extracted using RIPA buffer (Beyotime, Shanghai, China) containing protease and phosphatase inhibitors. The protein lysates were separated by 8%/10%/12% sodium dodecyl sulfate-polyacrylamide gel electrophoresis (SDS-PAGE) and transferred to polyvinylidene fluoride membranes (Millipore, USA). After blocking with 5% milk for 1 h at room temperature, the membranes were incubated with primary antibodies overnight at 4 °C. After washing, the membranes were incubated with the corresponding secondary antibodies conjugated to horseradish peroxidase. The membrane signals were detected using an enhanced chemiluminescence (ECL) kit (Pierce, USA). Antibodies used are listed in Table S4. For

immunoprecipitation, IgG or the specific antibody was incubated with the cell supernatant (lysed using NP-40 Lysis Buffer (Beyotime, Shanghai, China) containing protease and phosphatase inhibitors) overnight at 4 °C. The cell extracts were then immunoprecipitated with protein A/G magnetic beads (Bbimake, China) according to the manufacturer's instruction. Mass spectrometry (MS) analyses were performed by Wininnovate Bio (Shenzhen, China).

Immunofluorescence assay

Huh7 and SMMC-7721 cells were seeded on coverslips for the indicated transfections. Following incubation for 24-48 h, the cells on the coverslips were washed with phosphate-buffered saline (PBS) three times and fixed with 4% paraformaldehyde for 15 min, and then permeabilized with 0.25% Triton for 5 min. Thereafter, the cells were incubated with anti-pP65 (#3033, CST, USA) antibodies at 4 °C overnight. Following washes, the cells were incubated with rhodamine-conjugated goat antibodies against rabbit IgG (Abcam, Cambridge, UK) at 37 °C for 1 h. The nuclei were stained with DAPI (Abcam, Cambridge, UK) and the cell membranes were stained with Dil (#C1036, Beyotime, Shanghai, China) at 37 °C for 20 min. The images were captured with a BX51 or BX63 microscope (Olympus, Japan).

Dual-luciferase reporter assay

SMMC-7721 cells stably overexpressing HRG and the corresponding controls were cultured in 24-well plates and co-transfected with a NF-κB luciferase reporter. At 48 h post-transfection, luciferase activity was measured using the Dual-Luciferase Assay Kit (Promega, Madison, WI) according to the manufacturer's instructions.

Quantitative real-time PCR (qRT-PCR)

Total RNA was extracted from HCC cells or human tissues using TRIzol® reagent

(Invitrogen, Carlsbad, CA, USA) following the manufacturer's instructions. PrimeScript™ 1st Strand cDNA Synthesis Kit (TaKaRa, Tokyo, Japan) was used for first-strand cDNA synthesis. Real-time PCR was performed using SYBR® Green PCR kit (TaKaRa, Tokyo, Japan). The primers sequences used for the amplification are shown in Table S4.

5-ethynyl-20-deoxyuridine (EdU) assay

The HCC cell lines transfected with different vectors were seeded into 96-well plates and cultured overnight. Briefly, cells were incubated with 50 μM EdU for 2 h. After EdU-DNA incorporation, the cells were fixed and stained using EdU Cell Proliferation Assay Kit (Ribobio, Wuhan, China) according to the manufacturer's instructions.

Flow-cytometry assay

HRG-overexpressing HCC cells and the corresponding controls were stimulated with different doses of 5-fluorouracil. After 48 h, the cells were digested with EDTA-free trypsin and resuspended to generate a single-cell suspension. Apoptotic cells were evaluated using Annexin-V-APC and 7-AAD Apoptosis Detection Kits (KeyGen BioTECH, Suzhou, China). The cells were then analyzed with a FACScan flow cytometer using the FlowJo software (Tree Star Inc., Ashland, OR, USA).

Gene set enrichment analysis (GSEA)

GSEA was used to search gene signatures that correlated with HRG expression in the TCGA dataset as previously described [25]. Briefly, patients were split into two groups according to the HRG mRNA expression in the TCGA cohort. The GSEA software (GSEA v. 2.0, http://www.broadinstitute.org/gsea) was utilized to analyze, annotate, and interpret enrichment results.

Supplementary Data

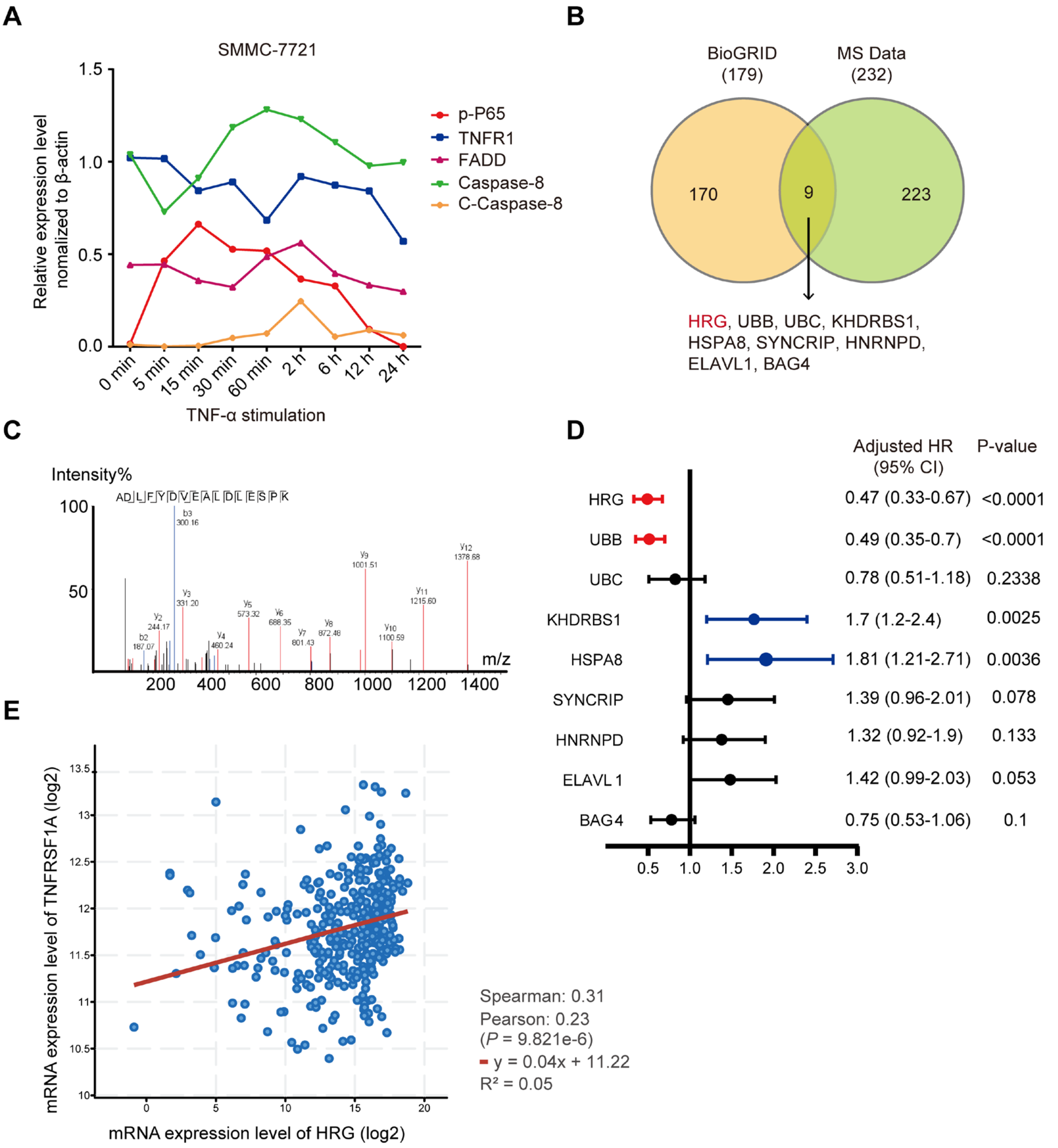

Figure S1. HRG is associated with TNFR1 in HCC. (A) Protein expression (normalized to β-actin) was measured at the indicated time points which matched those in Figure 1A. **(B)** Veen graph of TNFR1 interactors from BIOGRID database (376 interactors, https://thebiogrid.org/) and MS data (232 interactors). **(C)** The 55–70 kD band region was excised from the gel, and proteins were detected using mass spectrometry, whereupon the mass spectrum of HRG was detected. **(D)** Survival analysis of 9 genes with 95%CI and *P*-value were presented as Forest

plot. **(E)** Correlation between mRNA expression of TNFR1 and HRG in the TCGA cohort (http://www.cbioportal.org/).

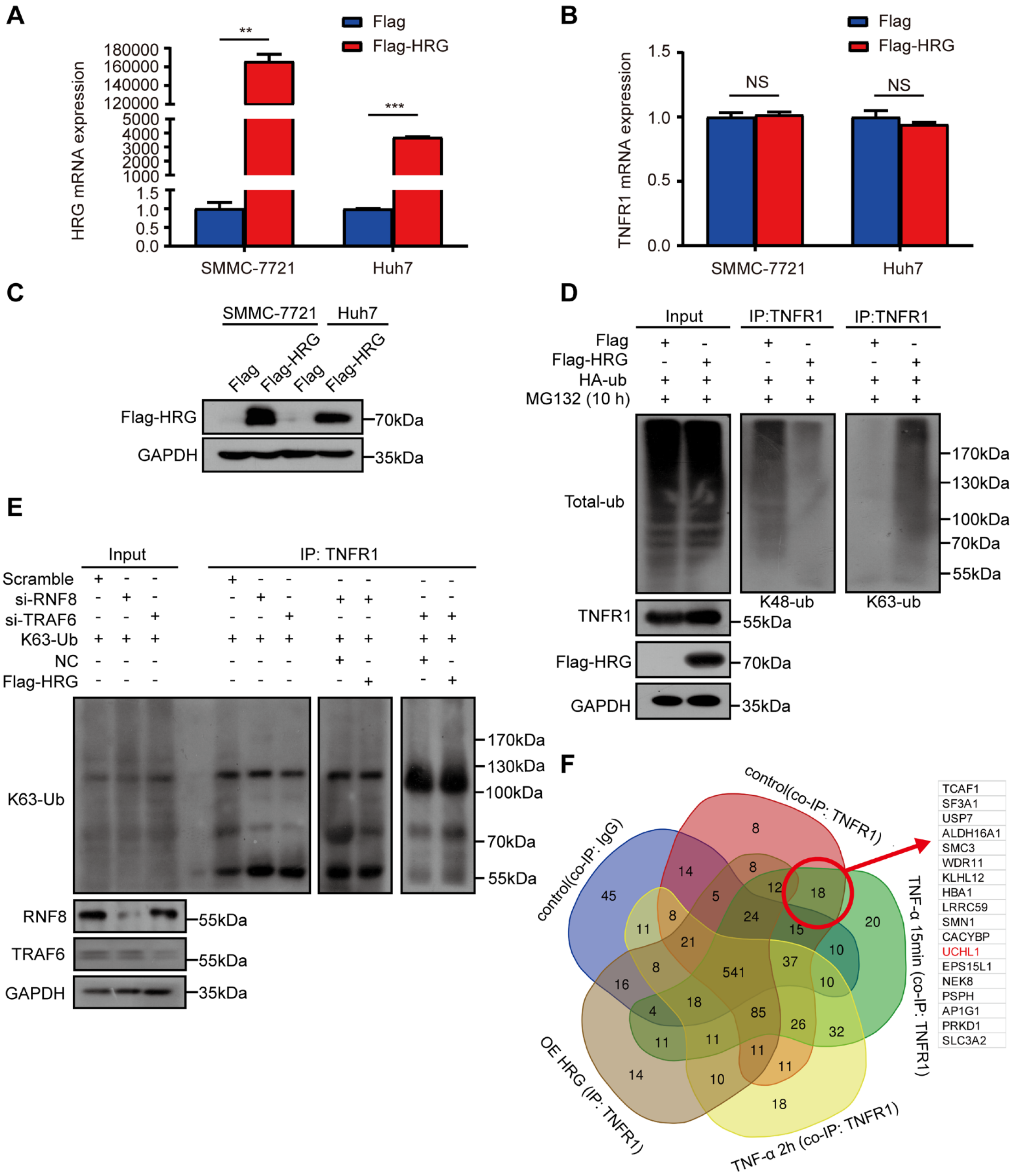

Figure S2. Upregulation of Lys (K)-63 ubiquitination of TNFR1 by HRG is RNF8- and TRAF6-dependent in HCC cells. (A-B) mRNA expression of HRG and TNFR1 was

analyzed in SMMC-7721 and Huh7 cells following overexpression of Flag-HRG. **(C)** Protein expression of Flag-HRG was detected in Flag-HRG overexpressed SMMC-7721 and Huh7 cells and the corresponding controls. (***, $P < 0.001$; NS = not significant). **(D)** Total and K48/K63-specific ubiquitination were detected in Huh7 HRG-overexpressing cells and controls after HA-Ub transfection (48 h) and MG132 treatment (10 μM). **(E)** K63-specific ubiquitination of TNFR1 of the following Huh7 cells (Scramble, si-RNF8, si-TRAF6, si-RNF8+NC, si-RNF8+Flag-HRG, siTRAF6+ NC and siTRAF6+ HRG) were immunoprecipitated with TNFR1 antibody and detected by western blot after HA-K63-ub transfection (48 h) and MG132 treatment (10 μM). **(F)** Veen graph of TNFR1 interactors from LC-MS analysis of TNFR1 co-IP lysates of different groups (control, TNF-α 15 min stimulation, TNF-α 2 h stimulation, and HRG overexpression).

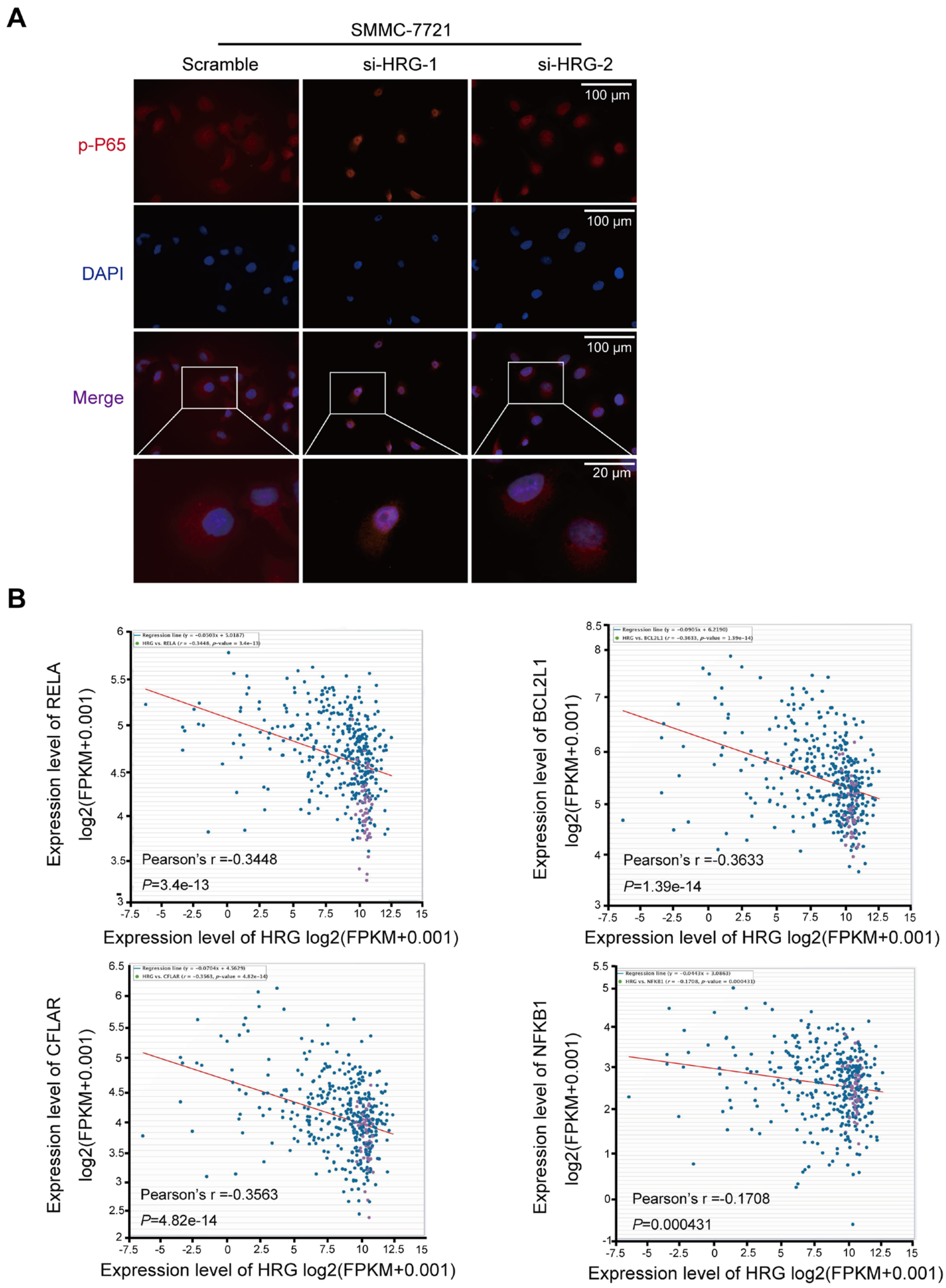

Figure S3. HRG negatively correlates with the NF-κB signaling pathway in HCC. (A) SMMC-7721 cells were analyzed by immunofluorescence for phospho-P65 (red) after transfection with either HRG or control siRNA. **(B)** Correlation between *HRG*, *RELA*, *BCL2L1*, *CFLAR*, and *NFKB1* were analyzed using ChIPBase v2.0.

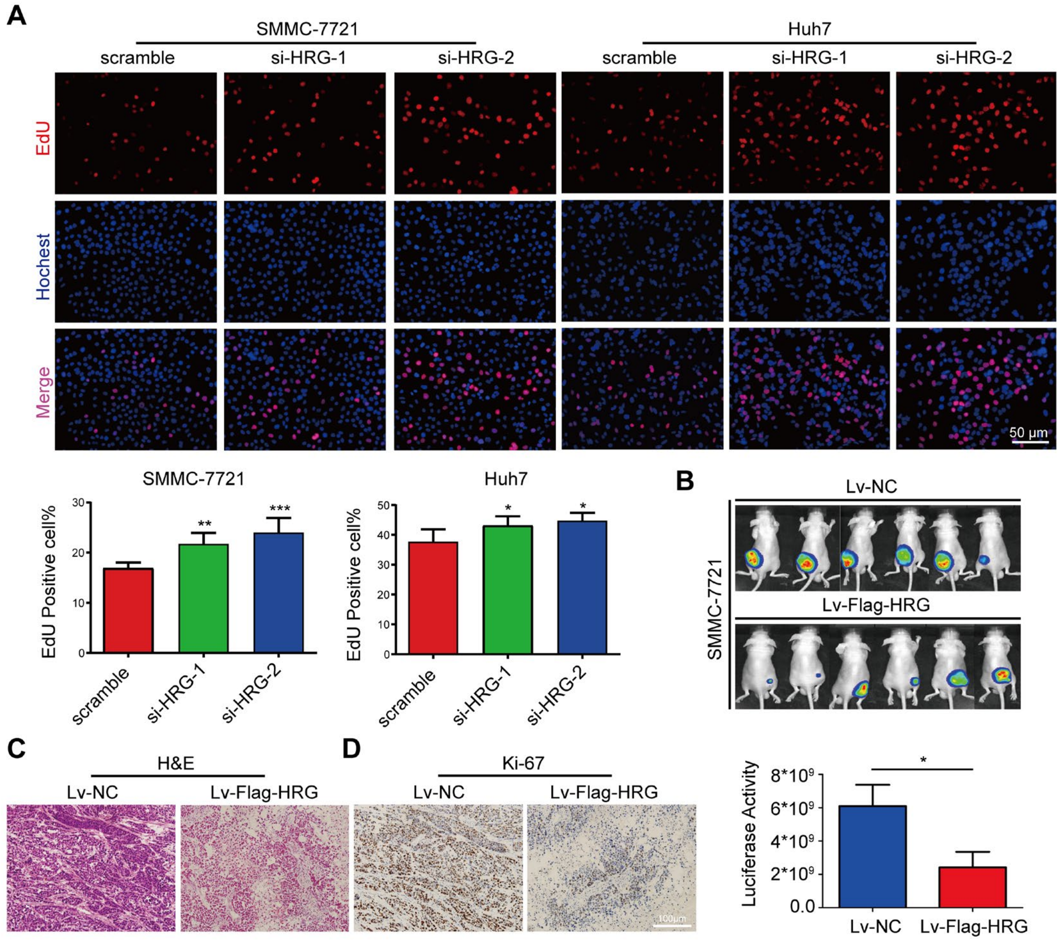

Figure S4. HRG inhibits tumor growth and promotes cell apoptosis. (A) SMMC-7721 and Huh7 cells were transfected with HRG siRNA, and cell proliferation was measured using the EdU assay. **(B)** Upper: Representative images of tumors formed in nude mice injected with the HRG transfected SMMC-7721 and control cells; Bottom: Fluorescence values of the two groups. **(C)** H&E staining of the subcutaneous tumors in nude mice. **(D)** Subcutaneous tumor tissues of nude mice were stained immunohistochemically using Ki-67 was stain. (*, $P < 0.05$; **, $P < 0.01$; ***, $P < 0.001$).

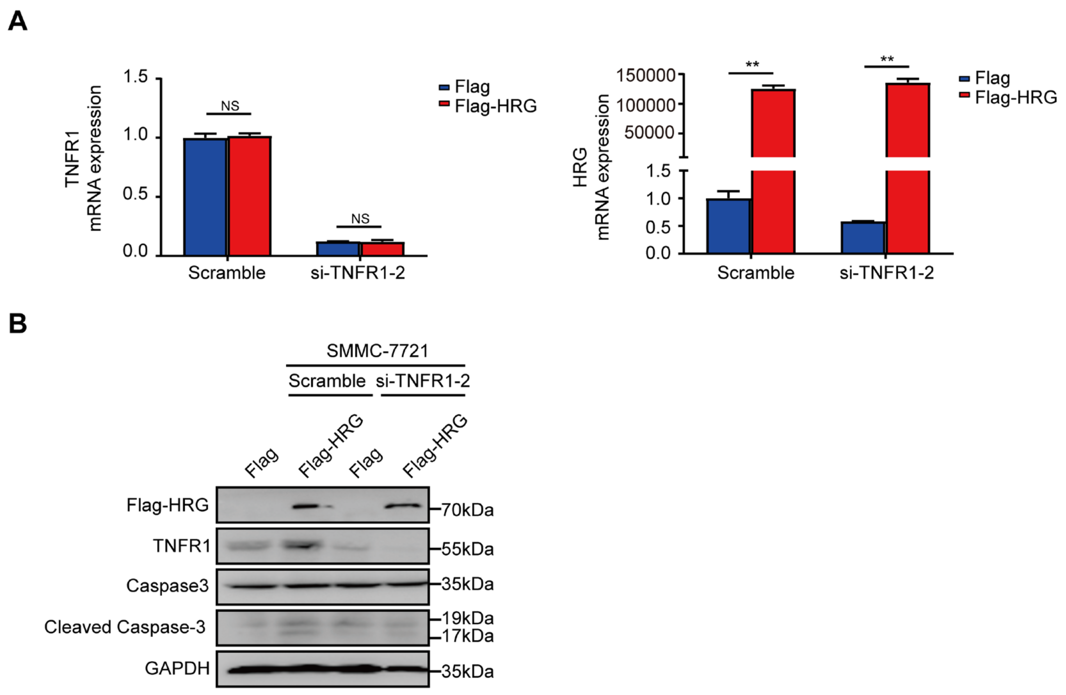

Figure S5. TNFR1 mediates the biological effects of HRG in HCC. (A) mRNA expression of TNFR1 and HRG in SMMC-7721 cells was analyzed using qRT-PCR following transfection with Flag-HRG and TNFR1 siRNA. **(B)** SMMC-7721 cells were transfected with Flag-HRG plasmid and TNFR1 siRNA, and then immunoblotted for the expression of Flag-HRG, TNFR1, Caspase3, and cleave-Caspase3 proteins. (*, $P < 0.05$; **, $P < 0.01$; NS = not significant).

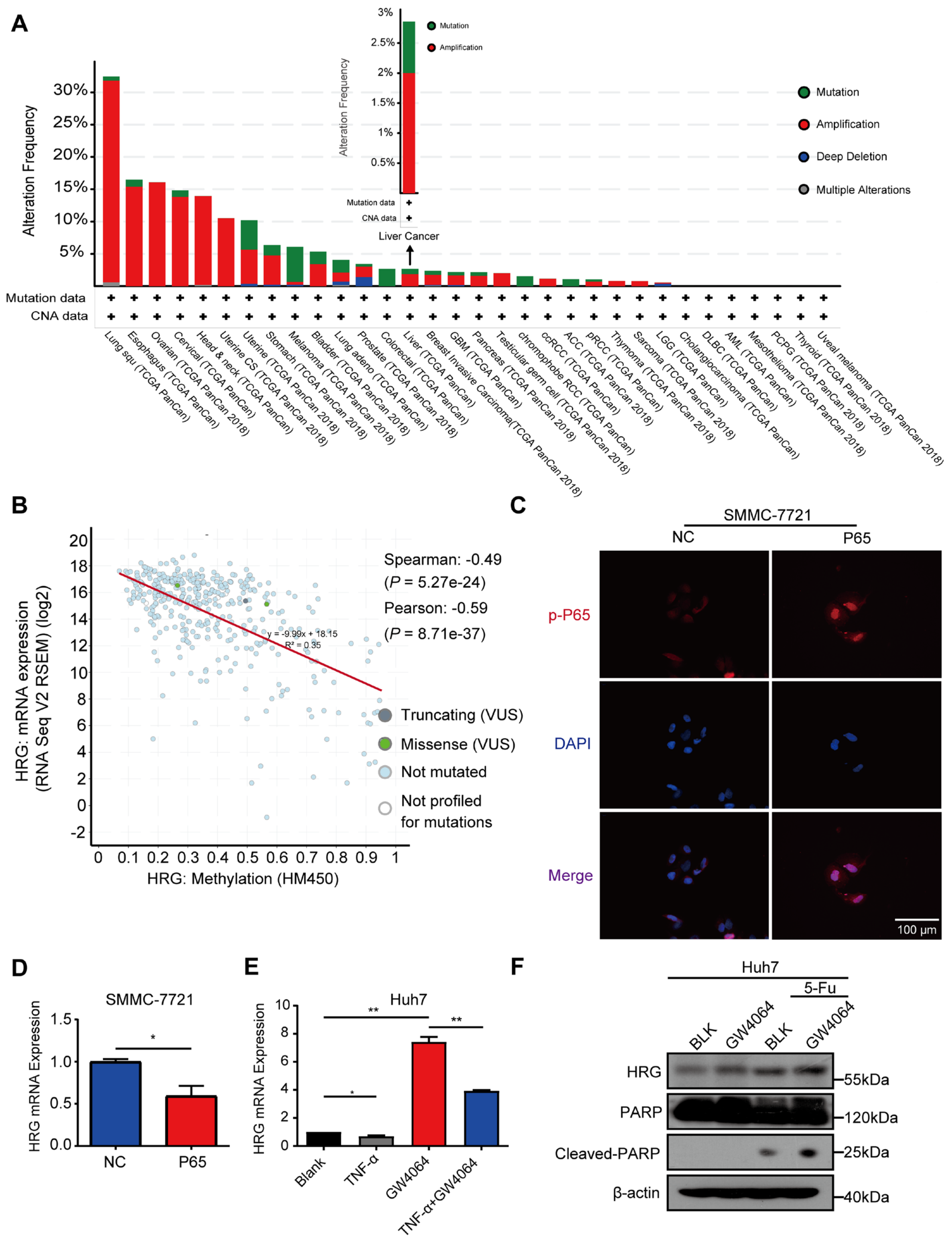

Figure S6. HRG expression is down-regulated in HCC and inhibited by NF-κB and DNA methylation. (A) Alteration in HRG gene: Data queried from the cBioPortal shows the alteration frequency of HRG gene in the different cancer types. Y-axis represents the alteration frequency of HRG gene (colors represent the type of alterations as shown in the figure). The

frequency of alterations is sorted on X-axis according to cancer types. **(B)** Negative correlation between the mRNA expression of HRG and HRG methylation (Pearson r = −0.59; $P < 0.001$). Line represents linear regression of data (y = −9.99x + 18.15; r2 = 0.35). **(C)** SMMC-7721 cells were analyzed by immunofluorescence for phospho-P65 (red) after transfection with either P65 plasmid or control vector. **(D)** HRG mRNA level in SMMC-7721 was analyzed using qRT-PCR following transfection with P65 plasmid (corresponding to Figure S6C). **(E)** mRNA expression of HRG in TNF-α-and GW4064-treated Huh7 cells was analyzed by qRT-PCR. **(F)** Expression of apoptotic protein was analyzed in Huh7 cells treated with 5-Fu and/combined GW4064 by western blotting. (*, $P < 0.05$; **, $P < 0.01$).

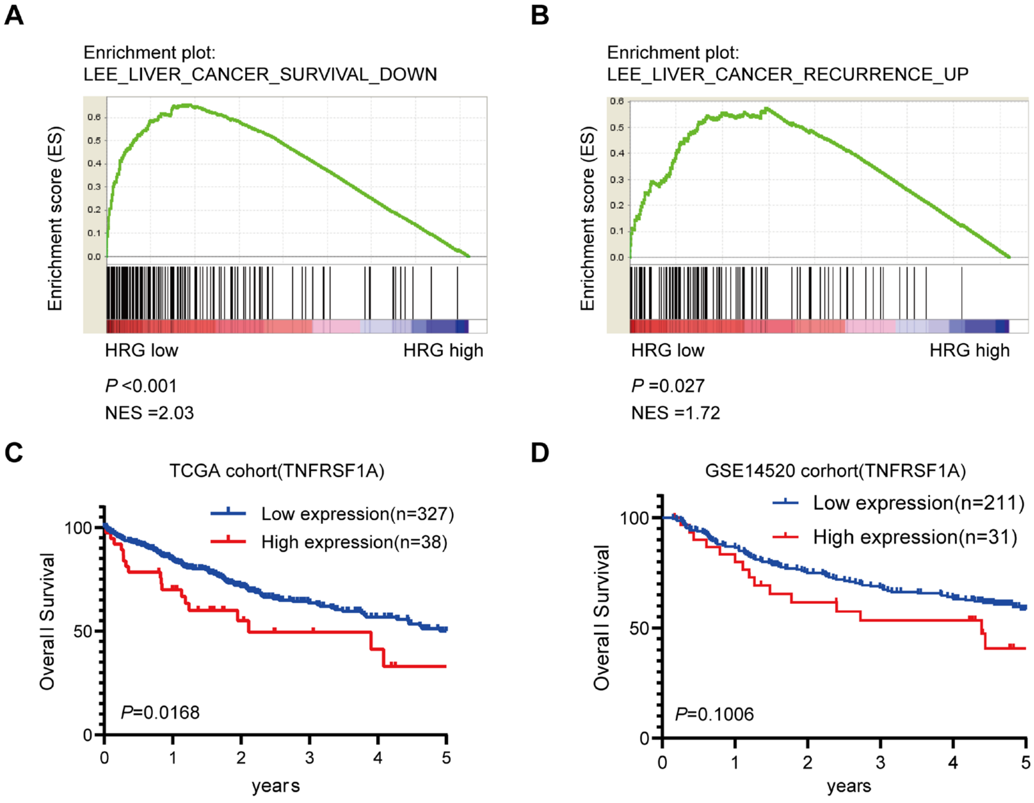

Figure S7. Correlation between HRG or TNFR1 expression and prognosis in HCC. (A-B)

Results of GSEA were plotted to visualize the correlation between the expression of HRG and gene signatures of liver cancer survival and recurrence in the TCGA cohort. **(C-D)** Kaplan-Meier analysis of overall survival in TCGA and GSE14520 cohorts based on TNFRSF1A expression status. X-tile software was used to generate optimal cut-off values and separate patients into high and low TNFRSF1A expression groups.
